# A Conversation With Lucy Kalanithi

**DOI:** 10.6004/jadpro.2016.7.7.2

**Published:** 2016-11-01

**Authors:** Pamela Hallquist Viale

I’m writing this editorial just a few days after the conclusion of our very successful, fourth annual JADPRO Live at APSHO conference that was held in sunny (at least for that week!) Washington, DC. Over 750 attendees enjoyed 4 days of focused, practice-changing information designed to educate and inspire the advanced practitioner. I know I came away with valuable insights on the management of major cancers and helpful tips on ways to improve the advanced practitioner’s ability to care for patients. We’ll be sharing some of these presentations on APSHO.org and advancedpractitioner.com. One of the highlights of the conference was the keynote presentation, "When Breath Becomes Air: A Conversation With Lucy Kalanithi," where I had the wonderful opportunity to sit down with Lucy and talk about her late husband’s book and legacy.

## THE MEMOIR

Dr. Paul Kalanithi thought he would spend the first 20 years of his life devoted to medicine and his last 20 years as a writer. In fact, his first educational degrees were in literature, as well as human biology and history and philosophy of science and medicine.

While he was finishing up a neurosurgery residency at Stanford University, his untimely diagnosis of lung cancer made it clear that his time was much shorter than anyone originally anticipated. He started crafting a beautiful memoir that became *When Breath Becomes Air*, a book that remained on *The New York Times* bestseller list for over 40 weeks.

In the first part of the book, Paul documents his journey as a neurosurgery resident. To the reader, his empathy and sensitivity toward patients as they navigate various diagnoses related to the brain and spinal cord rise clearly from the page.

Lucy is integral to the book. Paul weaves the story of their lives together, from medical students holding hands during lectures, to their mutual decision to have a child together, despite the fact that Paul wouldn’t be there to see their daughter grow up. When Paul underwent his therapies, Lucy, an internal medicine physician herself, was there to monitor and guide him, supporting him through the treatments.

Lucy also wrote an incredible epilogue to finish her husband’s story, describing her role as a witness as Paul faced his death with integrity. She joined us at the conference to share their story and to describe the things that were important to Paul and their lives together.

## OUR CONVERSATION AT JADPRO LIVE

The memoir, and in turn, Lucy’s conversation with the JADPRO Live attendees, was particularly devastating as the audience heard about the tremendous promise of this individual and his life ending much too soon. And yet, Paul’s incredible drive, passion, and love for his family and friends shine through so completely that one can’t help but feel inspired and lucky to have shared his experience within the pages of this remarkable memoir.

During our conversation, we discussed certain passages from the book, such as one that Paul wrote about the transition from physician and caregiver to patient. Suddenly, the act of living and planning their lives, careers, and future of their family together took a turn in the face of uncertainty. We also had a special moment to discuss the role of the advanced practitioner—an NP helping treat Paul—during his diagnosis. We ended the discussion with a quote from Paul to Cady, their daughter.

## CONCLUDING THOUGHTS

I feel very honored that we had Lucy join us for the conference. One of the most important lessons from this book is that as Paul examines his life and what is most important about it, he also forces the reader to look at his/her own life and how to live it. During our conversation, Lucy shared Paul’s unique humor with us and the story of their daughter Cady whom the book is dedicated to. This book showed the reader that living while dying, although not easy, can be achieved.

Lucy initially seemed surprised by the standing ovation the tearful audience gave her, but I wasn’t. Having Lucy Kalanithi join us for a keynote conversation about *When Breath Becomes Air* was a rare pleasure, and Paul’s memoir should become required reading in medical training. His memoir is exquisitely written and incredibly moving. His message of living each day as if it were the last is an important one. And if you haven’t read this book, I strongly recommend it.

